# Characteristic Variations and Similarities in Biochemical, Molecular, and Functional Properties of Glyoxalases across Prokaryotes and Eukaryotes

**DOI:** 10.3390/ijms18040250

**Published:** 2017-03-30

**Authors:** Charanpreet Kaur, Shweta Sharma, Mohammad Rokebul Hasan, Ashwani Pareek, Sneh L. Singla-Pareek, Sudhir K. Sopory

**Affiliations:** 1 Stress Physiology and Molecular Biology Laboratory, School of Life Sciences, Jawaharlal Nehru University, New Delhi 110067, India; ashwanip@mail.jnu.ac.in; 2 Plant Stress Biology Group, International Centre for Genetic Engineering and Biotechnology, New Delhi 110067, India; shweta2u@gmail.com (S.S.); rokebbrri@gmail.com (M.R.H.); sneh@icgeb.res.in (S.L.S.-P.); sopory@icgeb.res.in (S.K.S.); 3 Department of Plant Molecular Biology, University of Delhi South campus, New Delhi 110021, India

**Keywords:** glyoxalase pathway, glyoxalase III, methylglyoxal, multigene family, plants, abiotic stress tolerance, prokaryotes

## Abstract

The glyoxalase system is the ubiquitous pathway for the detoxification of methylglyoxal (MG) in the biological systems. It comprises two enzymes, glyoxalase I (GLYI) and glyoxalase II (GLYII), which act sequentially to convert MG into d-lactate, thereby helping living systems get rid of this otherwise cytotoxic byproduct of metabolism. In addition, a glutathione-independent GLYIII enzyme activity also exists in the biological systems that can directly convert MG to d-lactate. Humans and *Escherichia coli* possess a single copy of *GLYI* (encoding either the Ni- or Zn-dependent form) and *GLYII* genes, which through MG detoxification provide protection against various pathological and disease conditions. By contrast, the plant genome possesses multiple *GLYI* and *GLYII* genes with a role in abiotic stress tolerance. Plants possess both Ni^2+^- and Zn^2+^-dependent forms of GLYI, and studies on plant glyoxalases reveal the various unique features of these enzymes distinguishing them from prokaryotic and other eukaryotic glyoxalases. Through this review, we provide an overview of the plant glyoxalase family along with a comparative analysis of glyoxalases across various species, highlighting similarities as well as differences in the biochemical, molecular, and physiological properties of these enzymes. We believe that the evolution of multiple glyoxalases isoforms in plants is an important component of their robust defense strategies.

## 1. Introduction

**Figure 1:**
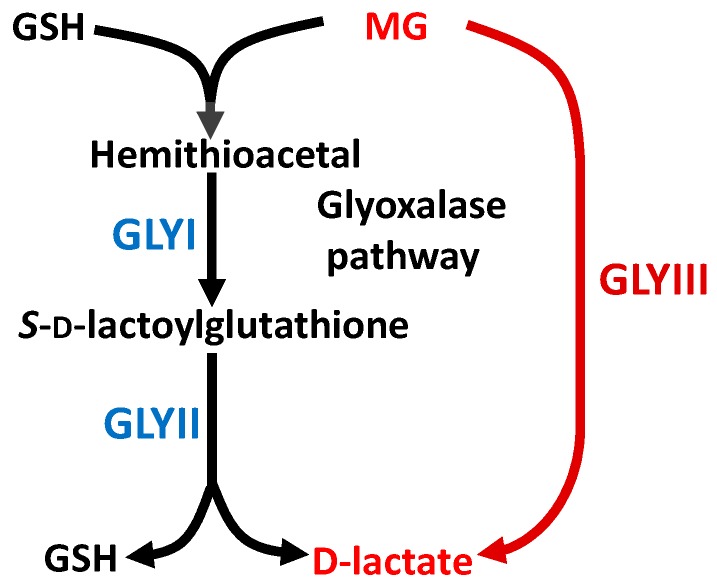
Glyoxalase pathway of living systems. The two-step glyoxalase pathway comprises glyoxalase I (GLYI) and glyoxalase II (GLYII) proteins, which catalyze the conversion of methylglyoxal (MG) into d-lactate using glutathione (GSH) as a cofactor, while glyoxalase III (GLYIII) proteins directly convert MG to d-lactate in a one-step reaction.

The research on methylglyoxal (MG) and the glyoxalase pathway was initiated more than 100 years ago and since then many discoveries have been made in the field of glyoxalases. MG is a highly potent glycating agent that can readily modify proteins, nucleic acids, and phospholipids [1,2]. Being generated as a byproduct of various metabolic reactions, primarily glycolysis, production of MG in living systems is inevitable. The glyoxalase pathway, by catalyzing the conversion of MG to d-lactate, detoxifies the cellular milieu [3] and hence is linked to many protective functions in both prokaryotes and eukaryotes (Figure 1). Besides the typical glyoxalase pathway that carries out the two-step metabolism of MG to d-lactate via glyoxalase I (GLYI) and glyoxalase II (GLYII) enzymes, living systems also possess a glyoxalase III (GLYIII) activity that directly converts MG to d-lactate without even requiring reduced glutathione (GSH) [4,5], otherwise needed as a cofactor for reactions catalyzed by the typical glyoxalase pathway (Figure 1). Proteins encoding GLYIII activity are members of a DJ-1/ThiJ/PfpI superfamily and possess a diverse range of functions besides GLYIII activity, such as chaperone activity (Hsp31) and protease activity (PH1704) [4,6,7]. In plants, their homologs have been termed DJ-1 (DnaJ-1) proteins, being similar in sequence and structure to human DJ-1. Importantly, glyoxalases are considered to be ubiquitous enzymes present in almost all living systems. However, studies on the microbial, animal, and plant glyoxalases reveal that these enzymes from different systems possess certain features specific to their kingdom.

GLYI, the first enzyme of the glyoxalase pathway, is a metalloenzyme requiring divalent metal ions, mainly Ni^2+^ or Zn^2+^, for activation. The metal ion requirement of the GLYI enzyme was previously believed to be linked to its origin, with the prokaryotic GLYI being Ni^2+^-dependent enzymes and the eukaryotic GLYI being Zn^2+^-dependent ones. However, with the advent of deeper knowledge into this field, the abovementioned classification was found to be invalid [8,9,10]. Interestingly GLYI, though not distinguishable based on the abovementioned metal activation properties, certainly differs in terms of its distribution, localization, and abundance patterns in prokaryotes, animals, and plants. Similarly, GLYII and GLYIII enzymes also possess variable characteristics in different species. Therefore, in order to highlight the differential characteristics of this MG detoxification pathway in various kingdoms, we discuss here the plant glyoxalase pathway in context of the distribution and localization patterns, biochemical properties, and physiological roles to bring out unique features of plant glyoxalases in comparison to microbial and animal glyoxalases. We therefore, describe how glyoxalases from different kingdoms are similar and yet different in terms of their biochemical and functional properties.

## 2. Metal Ion Dependence Properties of Glyoxalases

Like microbial and animal glyoxalases, plant glyoxalases metabolize MG, converting it to d-lactate through GLYI (*S*-d-lactoylglutathione lyase; EC 4.4.1.5) and GLYII (*S*-2-hydroxyacylglutathione hydrolase; EC 3.1.2.6) enzymes (Figure 1). In the first step, hemithioacetal, generated from the spontaneous reaction of MG with GSH, is converted to *S*-d lactoylglutathione (SLG) through GLYI [9]. The GLYII enzyme then converts SLG to d-lactate in the second step, recycling GSH in the process [11]. In conformity with the known metal ion dependent nature of microbial and animal glyoxalases, plant glyoxalases characterized from *Brassica juncea* [12], *Arabidopsis* [10], and rice [9] also show a metal ion-dependent nature, with BjGLYI from *B. juncea* and AtGLYI-2 from *Arabidopsis* being Zn^2+^-dependent enzymes, like other eukaryotic GLYI. However, recently characterized rice OsGLYI-11.2 enzyme and *Arabidopsis* AtGLYI-3 and AtGLYI-6 enzymes are Ni^2+^-dependent GLYI enzymes [9]. The presence of Ni^2+^-dependent GLYI in plants provides sufficient evidence to contradict the previous concept of linking Ni^2+^-dependent activation property of GLYI enzymes to prokaryotes [13] and lately to lower eukaryotes [14], thereby scrapping the previous metal-based classification of GLYI. In addition to the presence of Ni^2+^-dependent GLYI in rice, we have recently reported the presence of another GLYI (OsGLYI-8) in rice, which shows an apparently metal ion-independent nature and possesses several unique features not reported previously for any other GLYI enzymes [15]. OsGLYI-8 exhibits high GLYI activity despite the presence of only a trace amount of metal ions in its active site. However, the addition of metal ions in the growth medium during heterologous protein expression in bacteria results in the incorporation of one mole of metal ion (Zn^2+^ or Mn^2+^) per mole of OsGLYI-8 protein, which stimulates the GLYI activity of OsGLYI-8 by 20%–46% [15]. Similar mono-metalation has been observed for fully active GLYI from rice [9], *Escherichia coli* [16], and *Leishmania major* [14] enzymes. However, GLYI protein from humans binds two moles of metal ions per mole of dimeric proteins [17].

Like GLYI, GLYII enzymes also require divalent cations for their activity. GLYII proteins of prokaryotes and eukaryotes, including plants, usually possess a binuclear metal binding center, binding iron and zinc [18]. GLYII enzymes from rice (OsGLYII-2 [11]), and *Arabidopsis* (AtGLYII-2 [19] and AtGLYII-5 [20]) have been, likewise, found to possess a Zn/Fe binuclear center, similar to their homologs from *Leishmania infantum* [21] and humans [22]. Thus, no differences in the metal activation properties of GLYII enzymes from plants and other species have been reported so far.

Notably, the GSH-independent GLYIII activity (Hydro-lyase; EC 4.2.1.130) in all species displays a metal ion-independent nature [7,23]. The metal ions like Cu^2+^, Fe^2+^ and Zn^2+^ have been, in fact, shown to have inhibitory effects on the GLYIII activity of Hsp31 protein from *E. coli* [4]. The inhibition of GLYIII activity by Zn^2+^ occurs in a concentration-dependent manner, with approximately 10% at 25 µM and more than 50% at 100 µM. The authors proposed that the effects of metal ions on GLYIII activity might be due to a change in the oxidation state of the enzyme. By contrast, copper binding has been reported for human and *Arabidopsis* DJ-1 proteins; though not required for their GLYIII activity, it is essential for the activation of superoxide dismutase enzyme through copper transfer [24,25].

## 3. Presence of Glyoxalase Isoforms in Biological Systems

Microbial and animal genomes are known to encode a single copy of glyoxalases. However, as an exception, *Pseudomonas aeruginosa*, a proteobacteria, possesses multiple (three) *GLYI*, two encoding Ni^2+^-dependent forms and the third encoding a Zn^2+^-dependent form [8]. In prokaryotes, Ni^2+^-dependent GLYI forms are found to be more abundantly present in comparison to the Zn^2+^-dependent forms. This is possibly so because the Ni^2+^-dependent GLYI are considered to be primitive in origin, being vertically transferred during evolution from archaea, while the Zn^2+^-dependent GLYI are thought to have evolved later through acquisition via horizontal gene transfer events [26]. The first instance of the presence of Zn^2+^-dependent GLYI is found in the deltaproteobacteria *Bdellovibrionales*, an obligate parasite of Gram-negative bacteria that lacks Ni^2+^-transporters. It has been speculated that in the absence of Ni homeostasis in these organisms, Zn^2+^, which has similar properties to Ni^2+^, may have led to the evolution of Zn^2+^ specificity in GLYI [26]. Furthermore, humans and yeast also possess single *GLYI*, encoding Zn^2+^-dependent proteins [17,27]. Similarly, GLYII proteins are also encoded by single genes in the microbial and eukaryotic genomes (except plants and yeast), such as in humans [22], *E. coli* [28], and *L infantum* [21].

However, inspection of plant genomes reveals an altogether different distribution pattern of glyoxalases. A genome-wide study initially conducted in rice and *Arabidopsis* provided the first report on the presence of glyoxalases as multi-gene family, with rice genome possessing 11 *GLYI* and three *GLYII* genes while *Arabidopsis* genome coding for 11 *GLYI* and five *GLYII* genes [29]. Later, in silico analysis carried out on a large number of species belonging to different kingdoms demonstrated the presence of multiple glyoxalase isoforms as a general feature of plant genomes [26]. In addition, the study also suggested that not all genes annotated as putative glyoxalases possess glyoxalase activity, as predicted by the screening of protein sequences of glyoxalase genes for the presence of specific conserved metal ion and substrate binding sites. Furthermore, plant genomes were predicted to possess both Ni^2+^ and Zn^2+^ dependent GLYI, a feature unique to the plant kingdom [26]. The genome studies further revealed that among the multiple GLYI present in rice and *Arabidopsis*, it is the Ni^2+^-dependent form that is coded for by multiple genes, while only single gene codes for Zn^2+^-dependent GLYI [26,29]. Recently, a genome-wide study carried out in *Glycine max* further confirms the presence of glyoxalases as multi-gene family in plants, with *G. max* genome possessing 24 *GLYI* and 12 *GLYII* genes, of which three genes possibly code for Zn^2+^-dependent GLYI and eight code for Ni^2+^-dependent forms [30]. The presence of multiple Zn^2+^-dependent forms in *G. max* is probably the result of whole-genome duplication events in *G. max*. Nevertheless, these studies show that the Ni^2+^-dependent forms of GLYI are present in greater numbers in plant genomes as compared to the Zn^2+^-dependent forms. Table 1 shows the number of isoforms of GLYI and GLYII proteins present in different species, along with the number of respective metal ion-dependent forms of GLYI present in each genome.

Besides GLYI and GLYII, GLYIII activity in plants is also encoded by multiple genes. Recent studies in rice and *Arabidopsis* have revealed the presence of six *DJ-1* genes in both rice and *Arabidopsis* genomes, encoding for, respectively, 12 and 11 proteins [5,23]. Five out of the six genes in *Arabidopsis* encode functionally active DJ-1 proteins, with AtDJ-1d exhibiting highest catalytic efficiency as compared to the rest [23]. Unlike prokaryotes and other eukaryotes except plants, which possess a single copy of *GLYI* and *GLYII* genes, GLYIII activity in most organisms is determined by multiple proteins exhibiting different biochemical activities. Specifically, the members of the DJ-1/ThiJ/PfpI superfamily comprising the Hsp31, DJ-1 and PfpI (*Pyrococcus furiosus* protease I) proteins possess GLYIII activity in addition to other biochemical activities like chaperone or protease activity [25,40], indicating the presence of multiple GLYIII isoforms in other species as well (Table 1). One such example is the *Saccharomyces cerevisiae* genome, which encodes a single DJ-1 protein but four homologs of Hsp31 that show GLYIII activity [38]. Similarly, the *Schizosaccharomyces pombe* genome contains six DJ-1 homologs, designated Hsp3101–Hsp3105 and Sdj1 [41], and the *E. coli* genome encodes four proteins, HchA, YajL, YhbO, and ElbB, which exhibit glyoxalase activity with different substrate specificities, optimal pHs, and metal effects [31]. A comprehensive survey of DJ-1 and Hsp31 proteins in a wide range of taxonomically diverse fungal species reveals that most fungal species lack DJ-1 proteins but Hsp31 proteins are widely distributed among phylogenetically distant fungal species [42]. By contrast, in metazoans, DJ-1 proteins are ubiquitous whereas Hsp31 proteins are largely absent [42]. It can, therefore, be said that though DJ-1 are present as single proteins in prokaryotes and other eukaryotes except plants, GLYIII activity is encoded by multiple proteins in all species.

## 4. Domain Architecture of Glyoxalases

**Figure 2:**
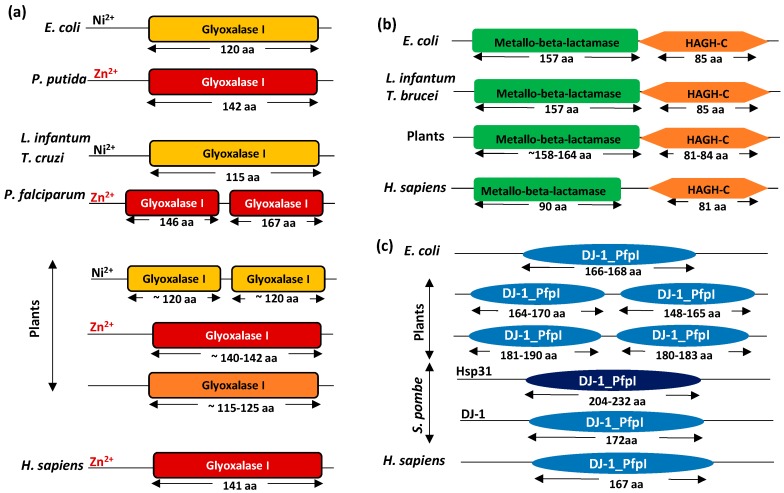
Different architectures of glyoxalase domains present in living systems. Schematic depiction of domains present in the (**a**) Glyoxalase I; (**b**) Glyoxalase II; and (**c**) Glyoxalase III/DJ-1 proteins across different kingdoms. Domain length has been indicated below each domain.

The domain architecture of plant glyoxalases is somewhat different from glyoxalases of other systems. Being present as multiple isoforms, glyoxalases, especially GLYI proteins, show various types of domain arrangements (Figure 2a). There are three main types of domain architectures observed in plant GLYI, as compared to the prokaryotic and other eukaryotic GLYI, which possess a single type (either Ni^2+^- or Zn^2+^-dependent) of GLYI domain (PF00903) (Figure 2a). In general, GLYI proteins are the members of a βαβββ superfamily of proteins [43] and their catalytic cleft is formed by the arrangement of the βαβββ motif either within one subunit, as in the monomeric *Plasmodium falciparum* GLYI [44], or at the interface of a homodimer, as in the case of *Homo sapiens* GLYI [22]. To date, all GLYI proteins characterized from prokaryotes have been found to be homodimeric, irrespective of their metal ion requirement, possessing two identical active sites at the dimer interface [22]. GLYI proteins from humans are also homodimeric but strictly require Zn^2+^ for activation [17]. In contrast, GLYI from *P. falciparum*, a protozoan parasite [44] and GLYI from yeast [36] have been found to be monomeric Zn^2+^-dependent proteins possessing two non-identical active sites (or two GLYI domains) in one polypeptide (Figure 2a). A closer investigation of the domain sequences of Ni^2+^- and Zn^2+^-dependent GLYI reveals that, generally, the Ni^2+^-dependent GLYI proteins possess shorter domain lengths (~120 amino acids), whereas Zn^2+^-dependent GLYI have domain lengths of ~ 140–145 amino acids due to the presence of (two [26] or three [45]) extra loop regions, otherwise missing in the Ni^2+^-dependent forms, which probably confers Zn^2+^ specificity to the GLYI proteins.

Similar to the presence of monomeric GLYI in yeast and *P. falciparum*, plants also possess monomeric GLYI (the first of the three types of domain architectures reported in plants), but these forms are Ni^2+^-dependent [9] (Figure 2a). Analysis of the biochemical properties of OsGLYI-11.2, a Ni^2+^-dependent monomeric GLYI from rice, suggests that out of the two possible active sites, only one is functionally active [9], which is not the case with yeast or *P. falciparum* monomeric GLYI [36,44]. The second type of domain present in the plant GLYI resembles the domains present in Zn^2+^-dependent forms of prokaryotic or eukaryotic homodimeric GLYI (Figure 2a). AtGLYI-2 from *Arabidopsis* and BjGLYI from *B. juncea* [12] possess this domain architecture and show a similar Zn^2+^-dependent activation profile [10]. However, its closest rice ortholog, OsGLYI-8, possesses similar domain architecture but differs in metal binding properties, showing apparently metal ion-independent nature with activity being stimulated by Zn^2+^ and Mn^2+^ [15]. The third type of domains present in the GLYI proteins of plants seems to be a mixed representation of the first two types of domains. These proteins possess a single GLYI domain (like Zn^2+^-dependent GLYI) but their domain length is shorter (~115–125 amino acids), characteristic of Ni^2+^-dependent GLYI enzymes (Figure 2a). Whether these proteins possess GLYI activity is not yet known.

Inspection of the amino acid sequences of GLYII proteins from different species reveals the presence of two different domains in every GLYII protein (Figure 2b). A metallo-β-lactamase domain (PF00753) is present in all members of the metallo-β-lactamase superfamily and is required for the catalytic activity, and a hydroxyacylglutathione hydrolase C-terminus (HAGH-C) domain, usually present at the C-terminus of GLYII proteins, forms the substrate binding site [30]. The length of these domains varies among different species (Figure 2b). Otherwise, though, there are no major differences in the domain architecture of GLYII proteins from different species.

However, studies on the DJ-1 proteins from rice and *Arabidopsis* reveal that plant DJ-1 proteins possess some distinguishing features in comparison to other species (Figure 2c). Unlike their homologs from humans and *E. coli*, all the *Arabidopsis* and rice DJ-1 genes have two tandem DJ-1/PfpI domains (PF01965), which may have been created by gene duplication events [5,23]. The modular sequences (i.e. two DJ-1/PfpI domain sequences) of these plant DJ-1 proteins are connected by a linker region of 7–18 amino acids, and have additional N- or C-terminal peptides of fewer than 55 amino acids, which are probably flexible and do not form a defined secondary structure [23]. The previous studies determining the evolutionary and functional relationships within the DJ-1 superfamily identified a sub-group of eukaryotic DJ-1 homologs that were most closely related to the bacterial *ThiJ* (*YajL*) gene [46]. The six *Arabidopsis thaliana* homologs were, however, not specifically clustered in this previous analysis, and were considered to be not belonging to the DJ-1 or ThiJ sub-groups. A further attempt to classify these *Arabidopsis* DJ-1 family proteins employed predicted active-site residues as a basis for comparison [47] and showed that the AtDJ-1d protein from *Arabidopsis* differed from its animal and bacterial homologs in respect of the configuration of its catalytic residues [23]. Also, unlike human DJ-1 and other homologs that are dimeric, some members of the *Arabidopsis* DJ-1/Pfp1 family are trimeric proteins [23]. Thus, the plant glyoxalases seem to have evolved additional features that may provide higher efficiency or better regulatory properties to these enzymes and, thereby, better adaptability to plants against their ever-changing environment, which they otherwise cannot escape.

## 5. Subcellular Localization Properties of Glyoxalases

Prokaryotic and human GLYI proteins are known to be present in the cytosol of the cells as their substrate MG is primarily produced in a glycolytic bypass [48]. However, some eukaryotic GLYI proteins have now been found to localize in other organelles as well. For instance, GLYI from *Leishmania donovani* localizes in the kinetoplast of the parasite apart from the cytosol [49]. Furthermore, prediction algorithms in combination with subcellular fractionation studies suggest that even *Trypanosoma cruzi* GLYI localizes not only to the cytosol but also to the mitochondria of *T. cruzi* epimastigotes [50]. Similarly, characterization of few *Arabidopsis* and rice GLYI proteins indicates that the plant genomes possess several GLYI isoforms that localize to organelles rather than the cytosol. Our previous in silico studies suggested that plant genomes probably possess chloroplastic, nuclear, and cytosolic forms of GLYI proteins [26]. Indeed, there is a predominance of chloroplast-localized GLYI proteins in plant genomes, as predicted by web-based localization prediction tools [26]. In *G. max*, out of the 13 possible active GLYIs, five are predicted to be chloroplastic and four code for cytosolic proteins. In addition, the authors predict three GLYI enzymes to be nucleus-localized [30]. In agreement, as we have reported in our recent studies, the characterization of nuclear localized GLYI proteins from both rice and *Arabidopsis* [15]. Our studies show that the nuclear localized GLYI enzymes are required for nuclear MG detoxification and the loss of nuclear AtGLYI-2 protein from *Arabidopsis* results in MG sensitivity [15]. The observed nuclear- and cytosol/chloroplast-based localization of plant GLYI has been mapped to Zn^2+^- and Ni^2+^-dependent forms, respectively, suggesting different modes of acquisition of these forms of GLYI enzymes in plants [26].

In contrast, there are no major differences in sub-cellular localization patterns of plant and other eukaryotic GLYII proteins. GLYII enzymes in eukaryotes are localized in either the mitochondria or the cell cytosol. Interestingly, a single human *GLYII* gene has been shown to encode both cytosolic and mitochondrial forms of the enzyme [51]. The human *GLYII* can get transcribed into two distinct mRNA species, from its nine and 10 exons, respectively. The nine-exon-derived transcript encodes both mitochondrial and cytosolic GLYII, whereas the transcript deriving from 10 exons has an in-frame termination codon and, hence, only encodes the cytosolic form of the protein [51]. Further, the yeast genome possesses two *GLYII* genes, one of which encodes a cytosolic protein and the other codes for the mitochondrial isoform [37]. Similarly, in *P. falciparum*, there are two *GLYII* genes; one codes for the cytosolic protein whereas the other codes for an altogether differently localized protein, being present in the apicoplast of the parasite [52]. Like most prokaryotes and eukaryotes, the *Arabidopsis* genome also possesses both mitochondrial and cytosolic forms of GLYII proteins [20,53]. Previously, GLYI proteins were considered to be cytosolic; hence, it was intriguing to observe the mitochondrial nature of some GLYII isoforms. However, with the discovery of newer GLYI candidates, it is becoming clearer that glyoxalases are not strictly cytosolic proteins and that the substrate of GLYII enzymes is generated in various sub-cellular organelles. However, no mitochondrial GLYI has been reported so far. Nevertheless, d-lactate formed via the action of GLYII proteins is metabolized in the mitochondria, specifically in the mitochondrial inner membrane space by the action of d-lactate dehydrogenases [54]. Even d-lactate generated in the cytosol is transported to the mitochondria through specific transporters [55,56]. Thus, the presence of mitochondria-specific metabolism of a GLYII product, d-lactate, suggests that the mitochondria are one of the major sites of MG metabolism.

Like GLYI and GLYII proteins, GLYIII proteins also exhibit differential localization patterns in the living systems. Some members of the rice and *Arabidopsis* DJ-1 family have been predicted to be chloroplast-localized [5,23]. *Arabidopsis* AtDJ-1b and AtDJ-1c proteins localize in the plastids [24], while two members of the rice DJ-1 family, OsDJ-1D.1 and OsDJ-1D.5, are predicted to be mitochondrial proteins [5]. Further, AtDJ-1a from *Arabidopsis* has been found to localize to both the cytosol and the nucleus, whereas AtDJ1-d localizes exclusively in the cytosol, being involved in oxidative stress response [24]. In contrast, human DJ-1 is an integral mitochondrial protein that controls the mitochondrial dynamics [57]. The Hsp31 protein from *Saccharomyces cerevisiae* is also a cytosolic protein but it has been found to relocalize to the mitochondria under oxidative stress conditions in order to protect the organelle from stress [58]. In *S. pombe*, sub-cellular localization studies of few Hsp31 and DJ-1 proteins showed that two of the three proteins localize to both the nucleus and the cytoplasm and one is exclusively present in the cytoplasm [42]. Overall, the differential localization patterns of these MG-detoxifying proteins, specifically in plants, suggest their important role in curbing MG-mediated damage in the cell (reviewed in [3]).

## 6. Kinetics and Regulation of Glyoxalase Enzymatic Activity

Under physiological conditions, the reaction rates for the catalysis of MG by GLYI enzymes have been found to be near diffusion-controlled limits [59], making the glyoxalase pathway very efficient in limiting MG concentrations to lower levels. However, variations are observed in the catalytic efficiencies of the glyoxalase enzymes characterized from different species (Table 2).

In prokaryotes, Ni^2+^-dependent GLYI enzymes have generally been found to possess higher overall catalytic efficiencies than Zn^2+^-dependent forms, and even exhibit higher affinity for the substrate, as evident from their relatively lower *K*_m_ values (Table 2). However, it is important to note here that since these enzymes have been characterized under different experimental conditions, a true comparison of the catalytic rates cannot be made, but an impression can surely be drawn from these results. The three GLYI enzymes from *Pseudomonas aeruginosa* can, nonetheless, be compared in real time as they have been characterized under the same experimental setup and indeed indicate comparatively lower catalytic efficiency of Zn^2+^-dependent GloA3 enzyme as compared to the two Ni^2+^-dependent GloA1 and GloA2 proteins [8]. By contrast, in eukaryotes, an opposite trend in catalytic efficiencies of Zn^2+^- and Ni^2+^-dependent GLYI is observed (Table 2). The Zn^2+^-dependent GLYI enzymes from humans [63], *P. falciparum* [44], and plants, including rice [15] and *Arabidopsis* [10], have been found to possess greater catalytic efficiencies than the eukaryotic Ni^2+^-dependent GLYI, which are primarily confined to plants (Table 2). This observed discrepancy in the catalytic efficiency is possibly so because, in plants, despite the presence of two GLYI domains (in a single polypeptide) in Ni^2+^-dependent GLYI, only one is functionally active [9] in contrast to the Zn^2+^-dependent GLYI enzymes, which possess two functionally active sites. The presence of only one functional site in plant Ni^2+^-dependent GLYI probably leads to lower catalytic efficiency of these Ni^2+^-dependent forms. However, as exceptions, Ni^2+^-dependent GLYI proteins from the eukaryotic protozoan parasites, *L. major* [14] and *T. cruzi* [50] show catalytic activity comparable to eukaryotic Zn^2+^-dependent forms as these enzymes likewise possess two functionally active sites. However, these enzymes show specificity for unusual thiols, trypanothione and glutathionylspermidine, respectively, instead of GSH, making these enzymes attractive targets for drug design.

Notably, an important mode of regulation of enzyme activity is observed for a few GLYI proteins. OsGLYI-8 from rice and PfGlo1 from *P. falciparum* have been shown to exhibit biphasic kinetics with a low-affinity and high-affinity substrate binding component and, thus, possess two apparent values for *K*_m_ and *k*_cat_ [44], a property unusual for GLYI enzymes otherwise known to follow Michaelis–Menten kinetics. In *P. falciparum* PfGlo1, the two sites are functionally active with similar catalytic activities but different substrate affinities [44]. The authors propose that the hemithioacetal substrate acts as a positive homotropic allosteric regulator of the PfGlo1 enzyme, as substrate binding to the first active site induces a different conformation of the second active site, leading to an increase in *k*_cat_ but a lowering of substrate affinity in comparison to the conformation occurring in the absence of the allosteric regulator [44]. In contrast, the two sequentially identical functional sites of OsGLYI-8 enzyme have different catalytic activities and substrate affinities [15]. The biphasic kinetics observed in the homodimeric OsGLYI-8 is intriguing. It has, however, been speculated to be due to the presence of a possible inherent conformational asymmetry in the otherwise identical active sites of the enzyme, similar to that reported for *E. coli* or human GLYI enzymes [67,68,69], causing the two sites to adopt different conformations and hence exhibiting differential kinetics.

Comparison of the catalytic activities of GLYII enzymes from different species reveals similar efficiencies of the enzymes in these species, except for protozoan parasites *T. brucei* and *L. infantum*, which show relatively lower catalytic efficiencies (Table 2). Notably, the GloB (GLYII) enzyme from *Salmonella typhimurium* is differentially inhibited by its product GSH, depending on the bound metal ion [70]. This metal-dependent inhibition has been shown to occur in metal-enriched forms of the enzyme, and can be exploited as a mechanism to regulate enzyme activity. Similarly, rice OsGLYII-2 enzyme activity has also been shown to be regulated by GSH [11]. Along with GSH, the other reaction product, d-lactate, also regulates OsGLYII-2 activity. d-lactate shows a non-competitive mixed type of inhibition and GSH shows a competitive type of inhibition. This observed end-product inhibition of OsGLYII-2 activity by GSH is relevant since it can ensure a tight correlation between OsGLYII-2 activity and cellular GSH levels and thus proper redox balance in the cell [11].

Like GLYI and GLYII enzymes, DJ-1 proteins have also been characterized from various prokaryotic and eukaryotic systems. In comparison to the enzymes of the typical glyoxalase pathway, these GSH-independent GLYIII enzymes possess relatively lower catalytic efficiencies (Table 2). This is probably due to the lower affinity of these enzymes towards MG as compared to GLYI/GLYII enzymes and these differences in the substrate specificity may be crucial for determining the mode of action of the two alternate pathways of MG detoxification. Notably, plant DJ-1 proteins are catalytically more efficient in comparison to corresponding proteins from other species (Table 2). For instance, AtDJ-1d from *Arabidopsis* and OsDJ-1C from rice have been found to possess, respectively, 14-fold and 3-fold higher catalytic activities as compared to the human DJ-1 protein and also have higher substrate affinities towards MG. Further, heat-inducible molecular chaperones Hsp31 from *E. coli* [4] and yeast [58] have also been shown to possess robust GSH-independent GLYIII activity, but less than that reported for the plant DJ-1 proteins (Table 2). Overall, glyoxalases are highly efficient enzymes but their activities might vary across species.

## 7. Structural Variations in Glyoxalase Enzymes

Prokaryotic and human GLYI display a characteristic homodimeric quaternary structure in which each monomer encompasses two βαβββ domains that interact to generate a continuous eight-stranded β-sheet with the other domain present in the opposite monomer [71,72], thus forming two active sites, each located at the interface between the monomers. However, both the active sites in the homodimeric GLYI from *Clostridium acetobutylicum* are located within the single subunits [73]. Similarly, even GLYI from *Pseudomonas putida* forms an active site within the same polypeptide in its monomeric form [60]. Overall, the crystal structure of all dimeric GLYI enzymes is 3D domain-swapped at the N-terminal helix [71].

**Figure 3:**
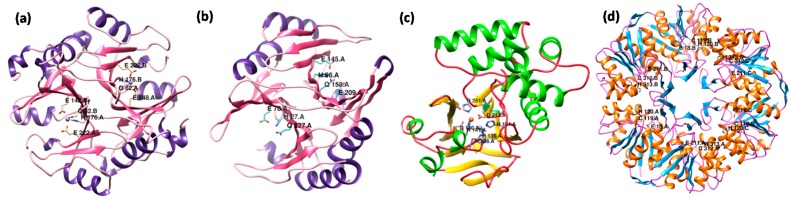
Three-dimensional homology model structure of rice glyoxalase proteins. Structures of (**a**) OsGLYI-8; (**b**) OsGLYI-11.2; (**c**) OsGLYII-3; and (**d**) OsDJ-1C proteins were built using a Swiss-model server (http://swissmodel.expasy.org/) based on the most similar structure available in the Protein Data Bank (PDB); human GLYI (1FRO) and *Zea mays* GLYI (5D7Z), *Arabidopsis* AtGLX2-5 (2q42) and *Arabidopsis* AtDJ-1d (3uk7), respectively. The conserved metal binding/active site residues have been indicated by ball-stick model. The structures were visualized using UCSF Chimera (http://www.cgl.ucsf.edu/chimera).

Structural analysis of Ni^2+^ and Zn^2+^-dependent forms of GLYI has revealed that only the enzymes in which the metal cofactor displays an octahedral coordination geometry are catalytically active [72,73,74]. Notably, Zn^2+^-dependent GLYI possess three short regions that are absent in the Ni^2+^-dependent GLYI. Deletional mutagenesis studies in Zn^2+^-dependent GloA3 protein from *P. aeruginosa* have revealed that the α-helix structural component, otherwise missing in the Ni^2+^-dependent GLYI enzymes, contributes significantly towards GLYI metal specificity, while the two small loop regions play a more crucial role in determining the magnitude of the enzymatic activity [74]. To date, the structure has not been solved for any Zn^2+^-dependent GLYI protein from plants. However, the homology modeling-based structure of a Zn^2+^-stimulated OsGLYI-8 protein from rice using human GLYI protein (PDB: 1FRO) as a template shows a similar arrangement of metal binding/active sites in OsGLYI-8, as observed for human GLYI (Figure 3a). In addition to the homodimeric forms, monomeric GLYI are also present in some organisms [9,36,44]. Recently, the crystal structure of a monomeric Ni^2+^-dependent GLYI has been deduced from *Zea mays* [62]. The ZmGLX1 enzyme comprises two structurally similar domains giving rise to two lateral concavities, with one site harboring a functional Ni^2+^-binding active site, while the function of the remaining cryptic active site remaining elusive, similar to that reported for OsGLYI-11.2 [9]. The three-dimensional structure of OsGLYI-11.2 protein generated using a Swiss-model server and *Z. mays* GLYI (PDB: 5D7Z) as template further indicates high structural similarity between the two proteins (Figure 3b).

Like GLYI, structural studies have also been carried out for GLYII enzymes from various species. Being members of the metallo-β-lactamase superfamily, GLYII enzymes contain the characteristic αβ/βα fold along with the conserved THxHxDH motif, able to bind up to two metal ions in their active sites [75]. The overall structure of GLYII can be divided into two parts according to its major secondary structure and fold (as also shown in Figure 2b), the β-sheet containing the N-terminal domain, and the α-helix containing the C-terminal domain [22]. The N-terminal domain resembles the protein fold similar to metallo-β-lactamase superfamily while the C-terminal domain is essential for substrate binding [22]. The crystal structure studies of GLYII proteins from humans, *L. infantum*, and *S. typhimurium* suggest that these proteins share similar fold and active sites [21,64]. However, the differential specificity of *L. infantum* GLYII towards trypanothione moieties as substrates is reflected as certain differences at their substrate binding site [21]. Further, the GloB enzyme from *S. typhimurium* has been found to accommodate different metal ions, though displaying similar efficiencies with different ratios of these metals [64], and is in contrast to the human GLYII enzyme, which possesses a di-Zn center [22]. This feature is indicative of the flexibility of the active site to accommodate any of these metal ions and can be accounted for by the presence of a conserved Asp residue in the GloB enzyme [64]. Furthermore, the crystal structure of AtGLX2-5 from *Arabidopsis* has also been deciphered and possesses structural features quite similar to that of the human GLYII [20]. However, EPR studies suggest that AtGLX2-5 contains multiple metal centers, including a predominant Fe^3+^-Zn^2+^ center and an anti-ferromagnetically coupled Fe^3+^-Fe^2+^ center, a feature not reported in human GLYII, thereby, demonstrating that the β-lactamase fold containing proteins can accommodate mixed metal centers. The structure of closest ortholog of AtGLX2-5 in rice, i.e., OsGLYII-3 protein, has been generated via homology modeling using AtGLX2-5 protein as a template and indicates high structural similarity between the two proteins (Figure 3c). Another GLYII-like protein from *Arabidopsis*, AtETHE1, has been structurally studied, revealing the presence of a fold that varies from the GLYII enzyme [34]. The removal of a two-helix bundle in AtETHE1 has been shown to result in the formation of a dimer interface that is missing from the GLYII enzymes. While the active site of AtETHE1 has enough space to fit the GSH group, there is substantially less space available for the other portion of the thioester owing to the extended C-terminal region in AtETHE1 that covers up much of the active site, leading to an altogether different reaction chemistry [34].

The structural properties of the third type of glyoxalase proteins (GLYIII) have been relatively less studied in plants, with the structure reported for only *Arabidopsis* AtDJ-1d protein [76]. The DJ-1/ThiJ/PfpI superfamily is an expanding family of proteins and includes various members, namely DJ-1, PfpI, and Hsp31. The crystal structure of human DJ-1 protein shows the presence of an α/β-fold, arranged similarly to the Rossman fold, and is conserved among the members of the DJ-1/ThiJ/PfpI superfamily [77]. The monomer of human DJ-1 is, in fact, structurally most similar to the monomer subunit of a protease I, PH1704, from *Pyrococcus horikoshii* [6]. However, the quaternary structures of PH1704 and DJ-1 are entirely different. The crystals of PH1704 show a hexameric structure (a trimer of dimers) roughly obeying 32 symmetry [6], in contrast to the dimeric association of DJ-1 [77]. Human DJ-1 contains an additional α-helix at the C-terminal region, which facilitates its dimerization, while its absence leads to hexamer formation in PH1704 [78]. The two monomers of the DJ-1 dimer are arranged in a head-to-tail fashion, such that the two active sites are on opposite sides of the dimer. Sequence comparisons suggest that all the PfpI family members (including PH1704) lack this helix, and are approximately 15 residues shorter than DJ-1 at the C terminus [77]. The putative active site comprising the Cys-His-Glu triad is present near the dimer interface [77]. Furthermore, the comparison of primary sequences reveals that the Hsp31 proteins are ~110 residues longer than DJ-1 and PH1704 proteins [78]. The crystal structure of *E. coli* Hsp31 shows three potential active sites, a hydrophobic patch for substrate binding, being responsible for chaperone activity of the protein; a potential protease-like catalytic triad comprising Cys^184^, His^185^, and Asp^213^; and a 2-His-1-carboxylate motif comprising His^185^, Glu^90^ and His^122^ [79]. Like Hsp31, human DJ-1 also possesses a hydrophobic patch, located in the shallow groove at the molecular interface, likely to be responsible for its chaperone activity [36], whereas Hsp31 contains this hydrophobic patch in each monomer [78]. The closest homolog of *E. coli* Hsp31 in yeast is the YDR533Cp protein, which shares the overall fold of the core domain with DJ-1 and PfpI proteins [80]. This protein contains catalytic triad analogous to that of Hsp31 and also an additional domain not seen in DJ-1 but present in Hsp31 and other members of the family. The cysteine in the catalytic triad (Cys-138) is oxidized in this crystal structure, similar to the modifications seen in human DJ-1. Like human DJ-1 and Hsp31, YDR533Cp appears to be a dimer but is formed by a different interface than that found in Hsp31 or other members of the superfamily [80]. Importantly, functional sites of DJ-1, Hsp31, and PH1704 proteins are present in the molecular interfaces or in the newly generated domains created in the quaternary structure but not in the DJ-1/ThiJ domain itself and thus oligomerization or generation of a new domain possibly serves as a strategy for creating various biochemical activities of DJ-1/ThiJ/PfpI family members [78]. The crystal structure of CaGlx3, the *Candida albicans* YDR533C/Hsp31 homolog, has also been solved and indicates that, unlike the homologous proteins from S. *cerevisiae* (YDR533Cp) and *E. coli* (Hsp31), CaGlx3 is a monomer [66]. As expected, the protein possesses a Cys-His-Glu catalytic triad, as found in other members of the Hsp31 clade.

Further to the recent availability of a structure of a plant DJ-1, AtDJ1-d from *Arabidopsis*, a two DJ-1/PfpI domain-containing protein, has provided new insights into the structural properties of DJ-1 proteins [76]. AtDJ-1d is trimeric with threefold symmetry, and this trimeric ring structure is stabilized by intra- and intermolecular interactions. Each monomer of AtDJ-1d is found to contain two tandem DJ-1/PfpI domains (N- and C-domains) connected by a β-sheet linker. The unit domain of a monomer comprises a core of six β-sheets and six α-helices, and is structurally similar to the human DJ-1 protein. The Cysteine residues, C^120^ and C^313^, important for glyoxalase activity, are contained in the N- and C-domains of AtDJ-1d, respectively, which are adjacent to each other at the intermolecular interface [76]. Similarly, the three-dimensional structure of rice OsDJ-1C protein generated through homology modeling studies using AtDJ-1d as a template indicates that these residues are conserved even in the OsDJ-1C protein (Figure 3d).

Further, structural studies on *Arabidopsis* AtDJ1-d and human DJ-1 proteins covalently bound to glyoxylate, an analog of MG forming a hemithioacetal that apparently mimics an intermediate structure in the catalysis of MG to lactate, also provides insights into the stereospecific mechanism of DJ-1 glyoxalases [76]. As opposed to the GLYI enzymes, which require metal ions for stabilization of the cis-enediolate intermediates formed after deprotonation of the substrates, DJ-1 glyoxalases possess a His residue for stabilizing the intermediate, which also enables stereospecific protonation as the catalytic reaction of DJ-1 glyoxalase requires a specific form of substrate (*R*-hemithioacetal) [76]. Overall, this multi-functional family displays wide variations in their structure owing to their various biochemical functions, but little is known as of yet about plant DJ-1 proteins.

## 8. Physiological Role of Glyoxalases in Living Systems

MG, being a potent glycating agent results in protein and DNA modifications, causes various disorders in animal systems and abiotic stresses in plants. Glyoxalase enzymes, by metabolizing MG, protect living systems from its deleterious effects. In *Caenorhabditis elegans*, glyoxalase expression has been linked to healthy aging [81]. Knockdown of *C. elegans* GLYI has been shown to increase the MG modifications of mitochondrial proteins and mitochondrial reactive oxygen species (ROS) production, thereby decreasing the *C. elegans* lifespan [81]. Decreased GLYI activity is, in fact, related to age-linked impairment of wound healing [82]; thus, increased MG is associated with several aging-linked diseases. In animals, a functional role of GLYI has been described in obesity, diabetes, cardiovascular, and chronic renal diseases (reviewed in [83]). Mice fed a high-fat diet with constitutive expression of GLYI siRNA, and thus GLYI deficiency, exhibit increased weight gain as compared to the wild-type controls [84], but show a decrease in weight gain in GLYI overexpressing transgenic mice [85]. Further, dicarbonyl stress caused by MG and other dicarbonyl compounds has been shown to possess dual functionality in cancer development and treatment. GLYI acts as both a tumor suppressor protein [86] and a mediator of multidrug resistance [87] in cancer chemotherapy. Interestingly, the discovery of *GLYI* gene duplication in some strains of mice has been linked with an anxiety phenotype [88]. MG is proposed to act as a mediator of sedation by agonizing the action of GABA_A_ receptor in primary cerebellar granule neurons; thus, increased *GLYI* copy number variation is associated with an anxiety phenotype [89].

In plants, the glyoxalase pathway has been primarily linked to stress tolerance mechanisms. MG levels in plants are known to rise under abiotic and biotic stress conditions [90,91], and the glyoxalase pathway, by detoxifying MG, provides stress tolerance in plants [92,93]. Plants overexpressing glyoxalase pathway genes have been shown to confer enhanced tolerance against salinity, heavy metal, and MG stresses, with plants transformed with both *GLYI* and *GLYII* genes performing better than those transformed with either *GLYI* or *GLYII* genes [92,93]. Likewise, tobacco leaves overexpressing TaGLYI from wheat showed increased tolerance to ZnCl_2_ stress as compared to control leaves [94]. In rice, the recently characterized glyoxalase genes OsGLYI-11.2 and OsGLYII-2 have also been shown to play a role in stress tolerance [9,11]. OsGLYII-2-overexpressing tobacco plants are able to adapt to salinity stress by maintaining better photosynthesis efficiency and anti-oxidant pool [11]. In *Arabidopsis*, loss of the *AtGLYI-2* gene, encoding a Zn^2+^-stimulated GLYI enzyme, has been shown to confer MG and NaCl sensitivity to the plants, with even lower doses of MG causing severe growth retardation in *Arabidopsis* [15]. Recently, the role of GLYI as a stigmatic compatibility factor has also been demonstrated in *Brassica napus* [95]. GLYI has been shown to be required for pollination to occur and is a target of the self-incompatibility system for preventing inbreeding or promoting hybrid vigor [95]. Suppression of GLYI resulted in reduced compatibility, and its overexpression in self-incompatible *B. napus* stigmas led to a partial breakdown of the self-incompatibility response. It is proposed that the degradation of GLYI after self-pollination concurrently increases MG levels and subsequent MG-mediated protein modifications (including that of GLYI), in turn triggering the MG-modified proteins to be efficiently targeted for destruction, leading to pollen rejection [95]. Furthermore, a GLYI protein, BnGLYI3 from *B. napus*, has been shown to be involved in seed thermotolerance, as its protein levels were significantly increased in response to heat stress and overexpression of *BnGLYI-3* gene imparted heat and cold tolerance in yeast [96]. Besides their role in stress tolerance, a few reports also suggest the role of the glyoxalase pathway in cell division. In fact, this function had been assigned to glyoxalases long before the discovery of their role in stress tolerance in plants. In 1983, Ramaswamy and co-workers had shown that rapidly proliferating cell lines exhibit increased GLYI activity [97]. The activity was found to be in good correlation with the mitotic index in *Pisum sativum* roots [97]. Similarly, in a *Datura* callus culture, the addition of mitotic inhibitors to the growth medium such as vinblastine were found to inhibit GLYI activity, whereas inducers of cell growth like spermidine enhanced GLYI activity [98]. Furthermore, both calmodulin inhibitors and lithium chloride, which inhibit cell proliferation, also reduced GLYI activity, as seen in *Brassica oleracea* [99].

The physiological role of the recently discovered class of novel glyoxalases, i.e., DJ-1 proteins, is largely unknown in plants. It is speculated that GLYIII activity is particularly important in conditions where the GLYI/GLYII system is less effective due to diminished levels of reduced glutathione, such as chronic oxidative stress, stationary phase, or sulfur limitation [66]. A homozygous GLYIII null mutant in *C. albicans* displays greater sensitivity to exogenous MG, higher levels of intracellular MG, and carbon source-dependent growth defects, especially when grown on glycerol [66]. Similarly, deletion of the *Hsp31* gene in *S. cerevisiae*, though it displays no apparent phenotype under standard growth conditions, makes the cells sensitive to temperature and a subset of ROS generators [100]. Recently, knockout studies in *S. cerevisiae* have revealed that the yeast Hsp31 mini-family plays a key role in the transition of proliferating yeast cells through diauxic shift into a non-proliferative stationary phase. Deletion of Hsp31 mini-family genes reduces chronological lifespan, impairs transcriptional reprogramming at diauxic shift, and impairs the acquisition of several typical characteristics of stationary phase, including autophagy induction [38]. Similarly, *S. pombe* homologs of DJ-1 are also stationary-phase-associated proteins involved in autophagy and the antioxidant defense in the stationary phase of *S. pombe* cells [41]. In humans, DJ-1 has been known to be involved in oxidative stress response and acts as a genetic cause for the early onset of Parkinson’s disease, its loss leading to neurodegeneration [101]. Since their discovery, various roles have thus, been assigned to DJ-1 proteins, primarily in protecting cells from oxidative damage [102]. In plants, not much is known about the role of DJ-1 proteins, but preliminary investigations on a few members of the *Arabidopsis* DJ-1 family reveal that AtDJ-1a, one of the GLYIII enzymes from *Arabidopsis*, is also involved in the oxidative stress response [24]. The loss of its function causes accelerated cell death in aging plants and transgenic plants overexpressing AtDJ-1a exhibit increased protection against environmental stress conditions. Superoxide dismutase 1 (SOD1) and glutathione peroxidase 2 (GPX2) have been found as the interaction partners of both AtDJ-1a and human DJ-1 proteins, and the authors showed that this interaction results in AtDJ-1a- and human DJ-1-mediated cytosolic SOD1 activation in a copper-dependent fashion [24]. Furthermore, another member of the *Arabidopsis* DJ-1 family, AtDJ-1c, was found to be required for viability [103]. Homozygous disruption of the *AtDJ-1c* gene leads to non-viable, albino seedlings and the plastids from the *atdj-1c* plants lacked thylakoid membranes and granal stacks, indicating that *AtDJ-1c* is essential for proper chloroplast development [103]. Thus, it can be said that, though glyoxalases play an important role in stress adaptation mechanisms in plants, it is quite possible that MG detoxification may be relevant in other physiological functions of living systems as well.

## 9. Functional Diversification in the Plant Glyoxalase Family

In addition to the presence of functionally active GLYI and GLYII proteins, the plant glyoxalase family also comprises a group of proteins that lack glyoxalase activity; these proteins are present in larger numbers than the functionally active glyoxalase proteins (Table 1). Among the plant glyoxalases, rice and *Arabidopsis* glyoxalase family members have been studied in greater depth [29]. Of the 11 *GLYI* genes present in rice and *Arabidopsis*, only four in rice and three in *Arabidopsis* are predicted to be functionally active [26]. Similarly in *G. max*, of the 24 genes annotated as putative GLYI, 11 are predicted to encode functionally active GLYI proteins [30]. The other seven GLYI members in rice, eight in *Arabidopsis*, and 13 in *G. max* possibly lack glyoxalase activity (Table 1). Previous literature also reports the presence of one such putative GLYI protein in the plant *Xerophyta humilis*, which has been annotated as a desiccation-induced-1^VOC^ (*dsi-1^VOC^*) gene [104]. *Xhdsi-1^VOC^*, along with its *Arabidopsis* ortholog, *AtGLYI-1* (*At1g07645*), has been shown to lack GLYI activity as it is unable to functionally complement the yeast GLYI mutant [104]. In desiccation-sensitive *A. thaliana*, *AtGLYI-1* is expressed at higher levels in mature seeds but is not detectable in vegetative tissues, even when they are exposed to abiotic stresses such as water loss, salt, or mannitol stress. In desiccation-tolerant *X. humilis* plants, *Xhdsi-1^VOC^* is abundantly expressed in seeds, roots, and leaves during a cycle of desiccation and plays an important role in allowing plants to bear the loss of ~95% of their relative water content [104].

Similarly, one of the three rice *GLYII* and two of the five *Arabidopsis GLYII* genes have been shown to encode inactive GLYII forms, indicating functional diversification of these proteins (Table 1). An AtGLX2-1 protein from *Arabidopsis*, despite showing extensive amino acid sequence similarity (88%) to the functionally active AtGLX2-5 protein, has been shown to lack GLYII activity [105] but possesses β-lactamase activity [35]. The presence of this activity in plants is intriguing as plants do not produce β-lactams. However, the authors propose that AtGLX2-1 may be an example of ongoing gene evolution, where duplication and functional divergence of an ancestral mitochondrial *GLYII* gene has led to the emergence of β-lactamase activity in AtGLX2-1. This is quite possible as AtGLX2-1 does not appear to be widespread in nature, being confined to only *Arabidopsis* and related crucifers [35]. A functional investigation of the role of AtGLX2-1 shows that its loss-of-function mutants and constitutively overexpressing plants resemble wild-type plants under normal growth conditions, but mutations in AtGLX2-1 inhibit plant growth and survival during abiotic stress [106]. The second *GLYII* gene from *Arabidopsis*, *AtGLX2-3*, which lacks GLYII activity, has in fact been shown to possess sulfur dioxygenase activity [107]. Unlike AtGLX2-1, which is not widespread, AtGLX2-3 has a ubiquitous nature, being present in a wide range of plant species including rice, humans, and other animals [107]. Even its rice ortholog OsGLYII-1 lacks GLYII activity [32]. Human ortholog of rice OsGLYII-1 and *Arabidopsis* AtGLX2-3 causes ethylmalonic encephalopathy (EE), a fatal autosomal recessive disorder characterized by early onset of EE and defective cytochrome C oxidase because of hydrogen sulfide accumulation in the bloodstream, severely damaging vascular endothelium and thereby causing the main symptoms of the disease [108,109]. Given the role of human ortholog in EE, these proteins have been named ETHE1 (Ethylmalonic encephalopathy protein 1). In *Arabidopsis*, ETHE1 is known to be critical for seed development and conditions that involve high protein turnover [107]. Since ETHE1 is a component of the mitochondrial sulfur catabolic pathway, its expression is upregulated in *Arabidopsis* under conditions that stimulate nutrient remobilization, such as prolonged darkness, drought, abscisic acid, germination, or unfavorable conditions leading to carbohydrate starvation [110]. In rice, *OsETHE1* has also been shown to be involved in abiotic stress response, being induced by high temperature, oxidative stress, and ABA and MG treatments [32].

Further, some members of the DJ-1 family in *Arabidopsis* have also been shown not to possess GLYIII activity. AtDJ-1c lacks GLYIII activity, using both MG and glyoxal as substrates, whereas AtDJ1-e and AtDJ1-f show only a trace amount of activity with MG and no activity with glyoxal [23]. AtDJ1-c is proposed to possess a role in plastid development and is expressed early in leaf development when chloroplasts mature, but is downregulated in older tissues [103]. In silico analysis reveals that the AtDJ-1c protein lacks a conserved Cysteine residue in the N-terminal domain, which is otherwise indispensable for activity [5]. Further, AtDJ-1c also lacks a conserved Cysteine residue in the C-terminal domain. There are other proteins that lack Cysteine at a similar position in the C-terminus, like AtDJ-1e and AtDJ-1f from *Arabidopsis* and OsDJ-1A and OsDJ-1F from rice [5]. In addition, OsDJ-1F, similar to AtDJ-1e and AtDJ-1f, also lacks the evolutionarily conserved histidine/tyrosine residue, indicating that OsDJ-1A and OsDJ-1F, may either completely lack GLYIII activity or show at least a partial loss of activity [5]. Overall, plant genomes have evolved multiple isoforms of glyoxalase proteins but some of these forms show functional divergence, suggesting an ongoing evolution of these proteins to acquire novel functions.

## 10. Conclusions

The glyoxalases family comprises multiple members in plants that are organized into various domain architectures and hence adopt different structures, leading to variations in the catalytic efficiency of these enzymes. By contrast, the presence of single forms of these enzymes in most prokaryotes and other eukaryotes does not allow much scope for variation in glyoxalase biochemistry, structure, or functional properties within these species. As plants cannot run away from a stress like animals, nature has likely accentuated these stress-tolerance or stress-recovery mechanisms, probably to allow for plants’ survival under unfavorable conditions.

## Figures and Tables

**Table 1:** Isoforms of glyoxalase enzymes present in different species.

Enzyme Source	GLYI				GLYII			GLYIII			
	Ni	Zn	Inactive	References	Active	Inactive	References	DJ-1	Hsp31	Classification Not Known	References
*Escherichia coli*	1	-	-	[13]	1	-	[28]	1	2	1	[4,31]
*Oryza sativa*	3	1	7	[9,26,29]	2	1	[11,32,33]	6	-	-	[5]
*Arabidopsis thaliana*	2	1	8	[10,26,29]	3	2	[19,20,29,34,35]	6	-	-	[23]
*Glycine max*	8	3	13	[30]	10	2	[30]	N.R.	N.R.		-
*Saccharomyces cerevisiae*	-	1	-	[36]	2	-	[37]	1	4	-	[38]
*Homo sapiens*	-	1	-	[17]	1	-	[39]	1	-	-	[7]

N.R. indicates “not reported”.

**Table 2:** Comparison of kinetic parameters of glyoxalase enzymes from various species.

Enzyme Source	Protein	Metal	*K*_m_ (µM)		*k*_cat_ (s^−1^)		*k*_cat_/*K*_m_ (M^−1^·s^−1^ × 10^6^)	References
**Glyoxalase I**								
*P. aeruginosa*	GloA3	Zn	287 ± 47		787		2.8	[8]
*P. putida*	GlxI	Zn	400 ± 200		500		1.25	[60]
*E. coli*	GlxI	Ni	27 ± 0.4		338		12	[16]
*Y. pestis*	GlxI	Ni	56 ± 0.6		306		5.5	[61]
*P. aeruginosa*	GloA1	Ni	32 ± 2		271		8.5	[8]
*P. aeruginosa*	GloA2	Ni	21 ± 0		247		12	[8]
*N. meningitidis*	GlxI	Ni	45 ± 5		204		4.5	[61]
*L. major*	GLO1	Ni	32 ± 3		800		25	[14]
*T. cruzi*	TcGLO1	Ni	8 ± 0.4		161		20	[50]
*P. falciparum*	PfGlo1	Zn	16 ± 3 *	103 ± 21 *	178 *	285 *	20.8 ± 2.9	[44]
*O. sativa*	OsGLYI-11.2	Ni	99.8		70.96		0.71	[9]
*O. sativa*	OsGLYI-8	Zn	4.3 ± 1 *	834 ± 172 *	22 *	178 *	36 ± 8	[15]
*A. thaliana*	AtGLYI2	Zn	786.78		137600		174.9	[10]
*A. thaliana*	AtGLYI3	Ni	45.32		728		16.08	[10]
*A. thaliana*	AtGLYI6	Ni	223.015		330		1.48	[10]
*Z. mays*	ZmGLX1	Ni	56.0 ± 5.0		N.R.		N.R.	[62]
*S. cerevisiae*	GloI	Zn	410 ± 40		1700		4.2	[36]
*H. sapiens*	GlxI	Zn	66 ± 5		1500		23	[63]
**Glyoxalase II**								
*E. coli*	GlxII	Zn	184 ± 22		53		0.47	[28]
*S. typhimurium*	GloB	Fe-Zn	241 ± 18		394.9		1.64	[64]
*T. brucei*	GLX2	N.R.	≥3000		4.5		0.0015	[65]
*L. infantum*	LiGLO2	Fe-Zn	324		3.52		0.0107	[21]
*O. sativa*	OsGLYII-2	Fe-Zn	254 ± 12		508.33		2.0	[11]
*O. sativa*	OsGLYII-3	N.R.	61		301		4.9	[33]
*A. thaliana*	AtGLX2-2	Fe-Zn	560 ± 143		564		1.0	[19]
*A. thaliana*	AtGLX2-5	Fe-Zn	391 ± 48		129		0.33	[20]
*S. cerevisiae*	GLO2	N.R.	112		979		8.7	[37]
*S. cerevisiae*	GLO4	N.R.	72.2		723		10	[37]
*H. sapiens*	GLX2	Fe-Zn	187		780		4.17	[39]
*Erythrocytes*	GLX2	N.R.	172		755		4.39	[39]
*Bovine liver*	GLX2	N.R.	190		4.37		0.023	[65]
**Glyoxalase III**								
**Enzyme Source**	**Protein**		***K*_m_ (mM)**		***k*_cat_ (min^−1^)**		***k*_cat_/*K*_m_ (M^−1^·min^−1^ × 10^5^)**	**References**
*E. coli*	Hsp31		1.43		156.9		1.1	[4]
*O. sativa*	OsDJ-1C		0.74		2500		33.6	[5]
*A. thaliana*	AtDJ-1a		5.48		102		0.19	[23]
*A. thaliana*	AtDJ-1b		4.16		154		0.37	[23]
*A. thaliana*	AtDJ-1d		0.1		1700		170	[23]
*S. pombe*	SpDJ-1		10.8		85.7		0.079	[42]
*C. albicans*	CaGlx3		5.5		468		0.85	[66]
*S. cerevisiae*	Hsp31		0.3854		150		0.578	[58]
*H. sapiens*	HsDJ-1		0.6		72.38		1.21	[7]

N.R. indicates “not reported”. * These enzymes show biphasic kinetics and thus possess two apparent *K*_m_ and *k*_cat_ values.
